# Astrocytes in stroke-induced neurodegeneration: a timeline

**DOI:** 10.3389/fmmed.2023.1240862

**Published:** 2023-09-07

**Authors:** Eileen Collyer, Elena Blanco-Suarez

**Affiliations:** Department of Neuroscience, Vickie and Jack Farber Institute for Neuroscience, Thomas Jefferson University, Philadelphia, PA, United States

**Keywords:** astrocytes, stroke, neurodegeneration, neuroprotection, neurorestoration, therapeutic targets

## Abstract

Stroke is a condition characterized by sudden deprivation of blood flow to a brain region and defined by different post-injury phases, which involve various molecular and cellular cascades. At an early stage during the acute phase, fast initial cell death occurs, followed by inflammation and scarring. This is followed by a sub-acute or recovery phase when endogenous plasticity mechanisms may promote spontaneous recovery, depending on various factors that are yet to be completely understood. At later time points, stroke leads to greater neurodegeneration compared to healthy controls in both clinical and preclinical studies, this is evident during the chronic phase when recovery slows down and neurodegenerative signatures appear. Astrocytes have been studied in the context of ischemic stroke due to their role in glutamate re-uptake, as components of the neurovascular unit, as building blocks of the glial scar, and synaptic plasticity regulators. All these roles render astrocytes interesting, yet understudied players in the context of stroke-induced neurodegeneration. With this review, we provide a summary of previous research, highlight astrocytes as potential therapeutic targets, and formulate questions about the role of astrocytes in the mechanisms during the acute, sub-acute, and chronic post-stroke phases that may lead to neurorestoration or neurodegeneration.

## 1 Introduction

Stroke is a debilitating condition caused by a sudden obstruction of cerebral blood flow resulting in reduced tissue perfusion to an area of the brain (Chen et al., 2014; Sommer, 2017). The World Stroke Organization currently ranks stroke as the third leading cause of death and disability worldwide (Feigin et al., 2022). Stroke can be ischemic, where the occlusion of a cerebral artery occurs due to a blood clot, or hemorrhagic, due to the rupture of a cerebral artery and subsequent bleeding into the brain parenchyma. Ischemic stroke is the most common (75%–80% of all strokes), while hemorrhagic stroke is associated with a higher mortality rate (van Asch et al., 2010; Saini et al., 2021; Feigin et al., 2022).

After a stroke, the tissue in the injury core sustains permanent damage caused by metabolic failure leading to massive cell death and inflammatory responses (Dobkin and Carmichael, 2016; Iadecola et al., 2020). The surrounding tissue, known as the peri-infarct area, becomes metabolically compromised by reduced blood flow and susceptible to the inflammatory response prompted by the injury. This triggers a multicellular response that can ultimately prime cells in the peri-infarct tissue to follow either cell death or survival pathways.

Injury progression after an ischemic stroke can be divided into three main stages: the acute phase (0–2 days post-injury in rodents, <2 months in humans), the sub-acute phase (2–30 days post-injury in rodents, 2–3 months in humans) and the chronic phase (>30 days post-injury in rodents, >3 months in humans) (Dobkin and Carmichael, 2016; Joy and Carmichael, 2021). The acute phase is characterized by massive cell death, onset of the inflammatory response, and astrocyte reactivity, ultimately leading to the formation of a border that delimits the lesion (Taylor and Sansing, 2013; Burda and Sofroniew, 2014; Li et al., 2022a). This stage is the target of early therapeutic interventions that aim to prevent acute or delayed cell death (Paul and Candelario-Jalil, 2021; Zhao et al., 2022). The sub-acute phase is characterized by spontaneous functional recovery underlined by endogenous mechanisms that may stimulate synaptic plasticity, dendritic spine remodeling, axonal sprouting, and brain map reorganizations (Murphy and Corbett, 2009; Benowitz and Carmichael, 2010; Pekna et al., 2012; Xerri et al., 2014). The sub-acute phase reflects the results of early interventions and is the target of most regenerative therapies (Cassidy and Cramer, 2017; Dhir et al., 2020). Finally, at the chronic phase, endogenous plasticity is greatly reduced, and functional impairments may be sustained if therapeutic interventions at earlier stages are ineffective (Wechsler et al., 2018; Dhir et al., 2020).

Astrocytes, through their various functions, are crucial to develop and maintain a healthy CNS and they are key players in many acute and chronic CNS diseases (Blanco-Suarez et al., 2017). They can provide structural and metabolic support, preserve the integrity of the blood-brain barrier (BBB), regulate the vascular tone in response to neuronal activity, mediate neuroinflammation, clear excessive neurotransmitters (glutamate homeostasis), balance oxidative stress and promote synaptic formation and maintenance (Liu and Chopp, 2016; Allen and Eroglu, 2017; Shen et al., 2021). Astrocytes are also crucial components of various multicellular complexes, such as the neurovascular unit and the blood-brain barrier (BBB), the tripartite synapse, and the glymphatic system (Jessen et al., 2015; Patabendige et al., 2021; Candelario-Jalil et al., 2022; He et al., 2022). Although astrocytes are more resilient than neurons to most stress conditions, their vulnerability increase in response to ischemic stroke (Giffard and Swanson, 2005; Qin et al., 2010) and their functions may be affected, aiding or hindering recovery throughout all the different post-stroke phases.

Several models, both *in vitro* and *in vivo*, have been developed to study stroke mechanisms and potential therapeutic approaches (Howells et al., 2010; Sommer, 2017; Li and Zhang, 2021; TuoZhang and Lei, 2022). *In vitro* stroke can be modeled through oxygen/glucose deprivation (OGD). In this model, cells are incubated in a glucose-free medium under deoxygenated atmosphere in order to mimic the conditions occurring during an ischemic stroke (Tasca et al., 2015). *In vivo* models can be broadly divided into two types: ischemic and hemorrhagic. Ischemic stroke models include global ischemia models, like 4-vessel and 2-vessel occlusion; and focal ischemic models, such as middle cerebral artery occlusion (MCAO), endothelin-1 occlusion and photothrombosis (Fluri et al., 2015; Li and Zhang, 2021). Hemorrhagic strokes have been modeled by intracerebral injection of either autologous blood or collagenase (Klebe et al., 2018). A large proportion of the current basic and preclinical research in stroke has focused on ischemic models due to its high prevalence in the clinic (van Asch et al., 2010; SainiGuada and Yavagal, 2021; Feigin et al., 2022).

**FIGURE 1:**
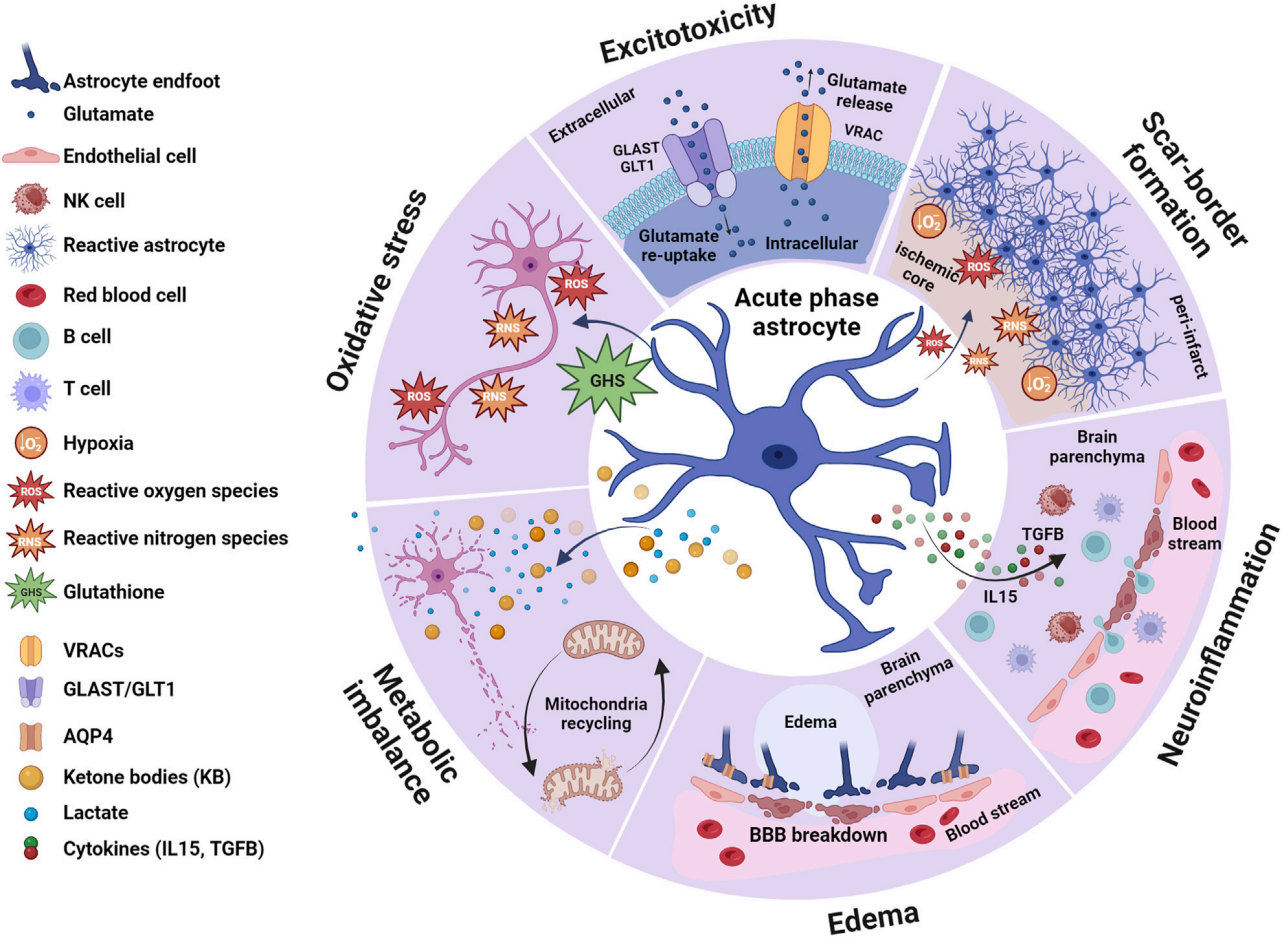
Astrocytes in the acute phase. During the earliest post-stroke phase, astrocytes contribute to different processes triggered by the ischemic signal, including excitotoxicity, oxidative stress, metabolic imbalance, edema generation, scar-border formation and neuroinflammation. Ischemia-induced glutamate release from neurons triggers the process known as excitotoxicity which in combination with oxidative stress and metabolic imbalance activates necrotic and apoptotic pathways, especially affecting neurons. Although astrocytes are not exempt of ischemic-induced cell death, they are less vulnerable than neurons to some of these processes, such as excitotoxicity. In physiological conditions, astrocytes keep extracellular glutamate levels balanced through the activation of GLAST and GLT1. However, during excitotoxicity, elevated levels of glutamate combined with changes in the ionic balance triggers reverse transport in GLAST and GLT1 further contributing to the extracellular glutamate accumulation. Similarly, activation of astrocytic VRACs exacerbates glutamate excitotoxicity. Ischemia triggers reactive astrogliosis due to the hypoxic conditions and the generation of ROS and RNS, and these reactive astrocytes are building blocks of the scar border that surrounds the core of the injury. In response to ROS and RNA, astrocytes may release GSH which protects neurons from oxidative stress. Astrocytes may also protect neurons by counteracting the metabolic imbalance created by the ischemic conditions, as they produce metabolic precursors such as lactate and ketone bodies (KB) and recycling mitochondria to restore ATP production. The metabolic imbalance elicited by the ischemic insult leads to cell swelling, interstitial fluid accumulation and generation of edema. Astrocytes are particularly vulnerable to cell swelling due to increased expression of the water channel AQP4 in their cell surface. This contributes to breakdown of the blood-brain barrier (BBB), which in combination with increased production of IL15 from astrocytes, allows peripheral immune cells (B and T cells) and NK cells to accumulate in the brain parenchyma. On the other hand, increased activation of astrocytic TGFβ signaling alleviates inflammatory response. In summary, astrocytes orchestrate both beneficial and detrimental cascades initially triggered in response to ischemic stroke, rendering them interesting targets to prevent detrimental outcomes at later post-stroke phases. Created with BioRender.com.

**FIGURE 2:**
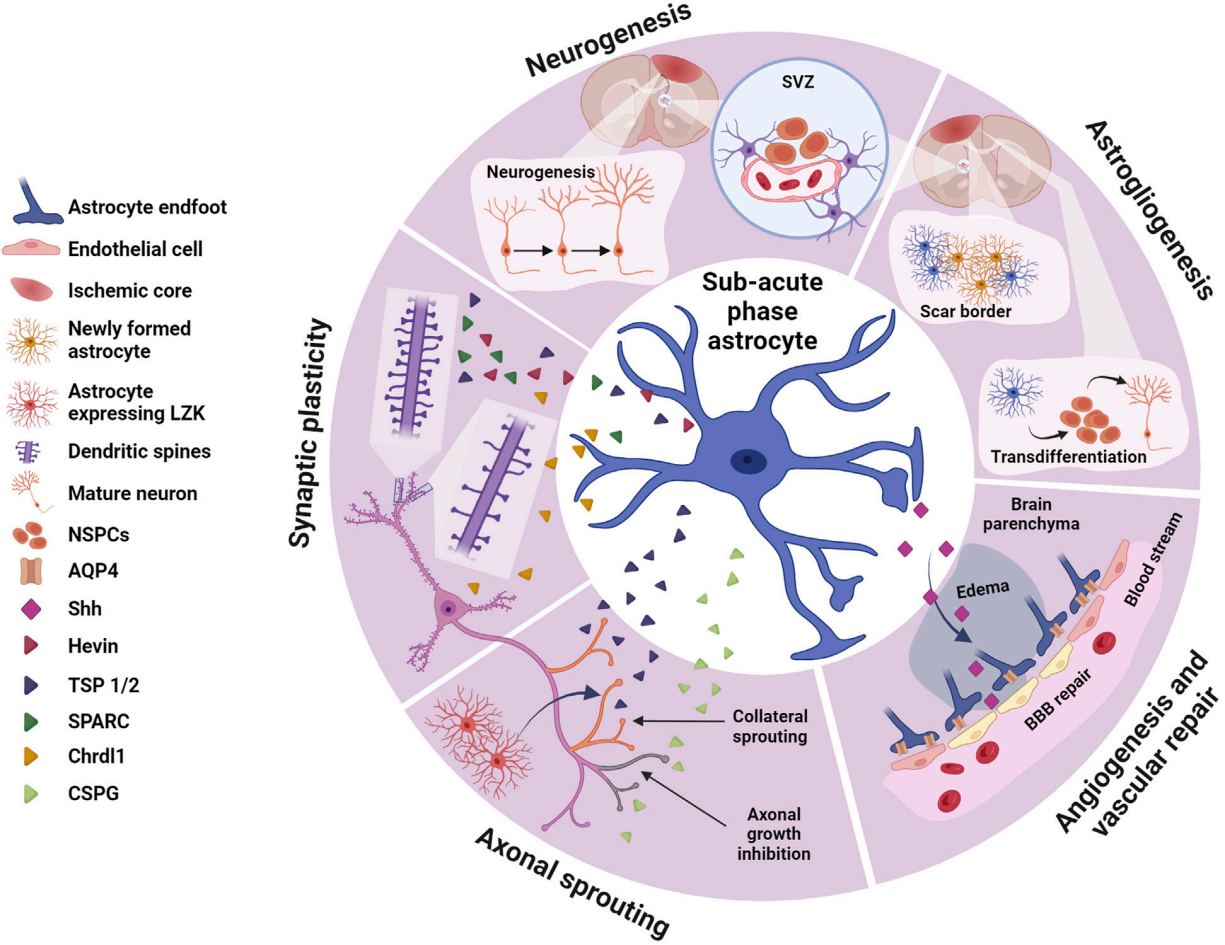
Astrocytes in the sub-acute phase. During the sub-acute phase of stroke astrocytes play crucial roles in neurogenesis and astrogliogenesis, synaptic plasticity, axonal sprouting and angiogenesis and vascular repair, potentially contributing to spontaneous functional recovery. Astrocytes, via different molecular cascades, stimulates neurogenesis in the SVZ increasing proliferation of NSPCs and their migration to the peri-infarct area. NSPCs may also transdifferentiate and contribute to astrogliogenesis, generating new astrocytes that integrate in the scar border. Via secretion of a variety of factors, astrocytes promote synaptic plasticity via dendritic spine and synaptic remodeling, as well as regulating axonal sprouting. Some of these astrocyte-derived proteins, such as hevin, SPARC or TSP 1 and TSP2, promote circuit remodeling by restoring synapses and dendritic spines. Others like Chrdl1 may inhibit dendritic spine plasticity. TSP 1 and TSP 2 also contribute to axonal sprouting, crucial in circuit remodeling and recovery from ischemic stroke, while other proteins produced by reactive astrocytes such as CSPGs inhibit axonal growth. Shh, another astrocyte-derived protein participates in post-stroke repair by facilitating angiogenesis and vascular repair and contributing to BBB repair. Created with BioRender.com.

**FIGURE 3:**
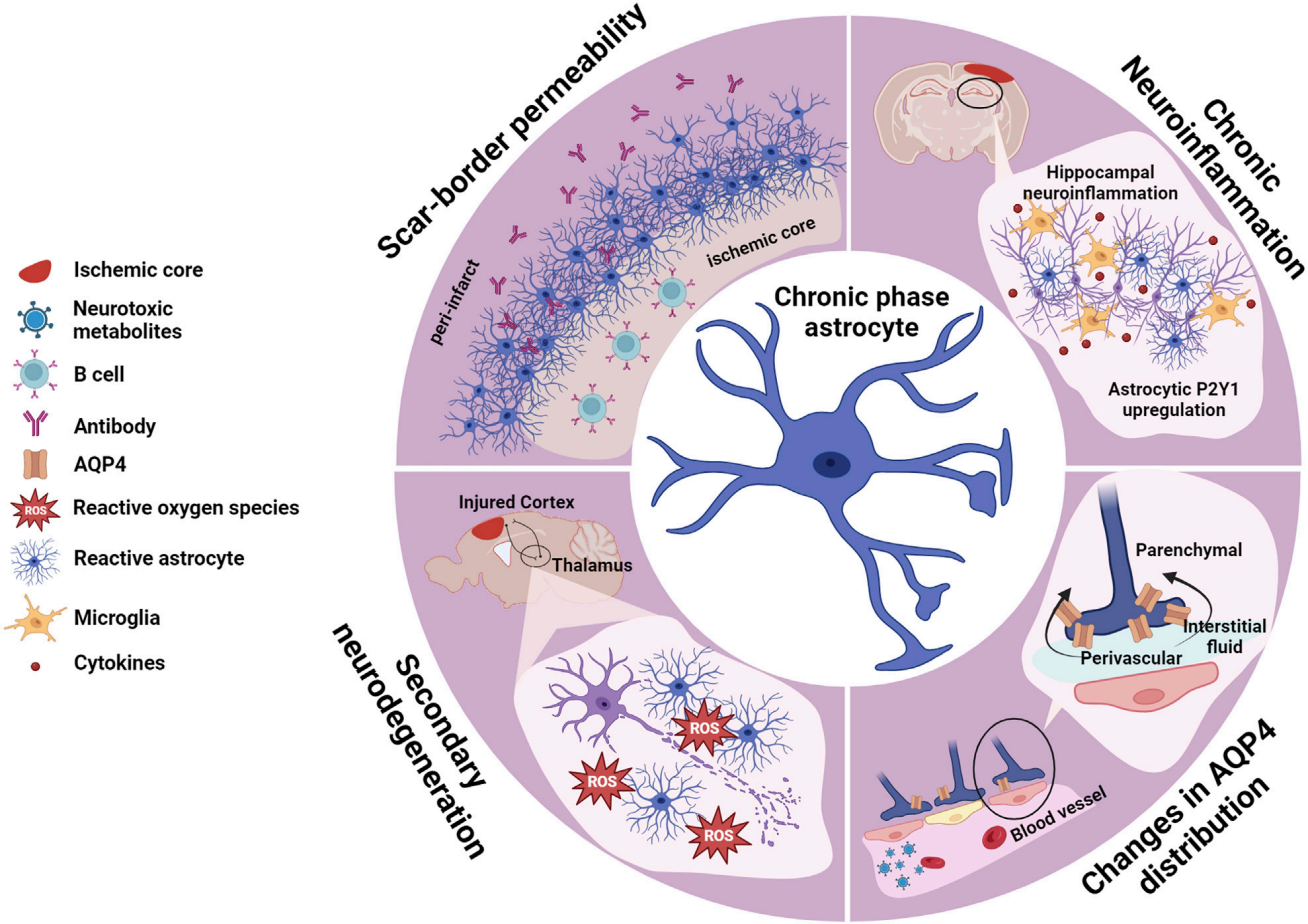
Astrocytes in the chronic phase. Consolidation of the scar-border and closure of the plasticity window (sub-acute phase) marks the transition to the chronic phase, where long-term consequences of tissue loss and adaptive and maladaptive remodeling manifest. Post-stroke cognitive impairment (PSCI) is one of the major burdens derived from ischemic injuries, and a few mechanisms have been identified as contributors to this condition. Focal ischemic stroke triggers a wave of secondary neurodegeneration, triggering ROS generation and affecting distant brain areas anatomically connected to the injured tissue but unaffected by the initial lesion. Chronic neuroinflammation and oxidative stress have been linked to this process. Neuronal loss in the hippocampus, which exacerbates cognitive impairment, is linked to the upregulation of the astrocytic receptor P2Y1. AQP4 distribution from perivascular to parenchymal within the astrocyte endfeet has been identified as one PSCI hallmark. Ischemic-triggered aberrant AQP4 redistribution has additional consequences impairing effective clearance of neurotoxic metabolites furthering cognitive deficits. The scar-border eventually becomes permeable which causes accumulation of antibodies in the peri-infarct area, which together with B cell infiltration into the ischemic core contribute to PSCI. Created with BioRender.com.

This review will explore the roles of astrocytes during the acute (Figure 1), sub-acute (Figure 2), and chronic phases (Figure 3) after ischemic stroke, which eventually may lead to neurodegeneration, highlighting recent advances toward potential therapeutic approaches.

## 2 Astrocytes in the acute phase

### 2.1 Oxidative stress

The mechanisms by which reactive oxygen species (ROS) and reactive nitrogen species (RNS) cause cerebral tissue damage are not well understood. ROS/RNS are reported to trigger a variety of molecular cascades that increase BBB permeability and alter brain morphology, causing neuroinflammation and neuronal death, events where glial cells such as astrocytes play important roles (Zhu et al., 2022a). Antioxidants are crucial in maintaining the oxidative balance in the brain, and thus, they have been proposed as promising targets to halt neurodegeneration and cognitive impairments (Franzoni et al., 2021).

Astrocytes are rich in glutathione (GSH) (Dringen et al., 1999; McBean, 2017), a crucial antioxidant that can reduce ROS toxicity and oxidative stress after ischemic insults (Mizui et al., 1992). *In vitro*, astrocytes can provide neurons with GSH (Wang and Cynader, 2000), and neurons co-cultured with astrocytes exhibit a higher tolerance to glutamate excitotoxicity through a GSH-dependent mechanism (Chen et al., 2001), suggesting that astrocytes protect neurons from oxidative stress by synthesizing and releasing GSH.

Nuclear factor erythroid-2 related factor (Nrf2), a transcription factor that regulates the expression of several antioxidant enzymes, is increased in neurons and astrocytes in the peri-infarct area in response to ischemic injury in mice and humans (Dang et al., 2012; Takagi et al., 2014). Upregulation of Nrf2 in astrocytes has been shown to be neuroprotective, via decrease of Nrf2 ubiquitination, reducing ROS levels after ischemia *in vitro* (Hong et al., 2023). Acute administration of Nrf2 activators after transient middle cerebral artery occlusion (tMCAO) *in vivo* upregulates the antioxidants superoxide dismutase (SOD) and glutathione peroxidase, reducing infarct volume and improving neurological function in mice (Takagi et al., 2014; Hong et al., 2023). This suggests that acute upregulation of astrocytic Nrf2 may protect neurons from ischemic-induced neurodegenerative processes. Nrf2 can be stabilized by DJ1, a protein deglycase also known as PARK7, by preventing its association with Keap1 and subsequent ubiquitination (Clements et al., 2006), driving the upregulation of GSH synthesis during oxidative stress (Zhou and Freed, 2005). DJ1 is abundantly expressed in peri-infarct reactive astrocytes during ischemic stroke in rodents and humans (Mullett et al., 2009; Yanagida et al., 2009; Peng et al., 2019). Overexpression of DJ1 in astrocytes protects neurons from OGD-induced cell death *in vitro* (Peng et al., 2019). Genetic ablation of DJ1 or lentiviral-mediated downregulation of DJ1 before cerebral ischemia results in increased infarct volume and neurological impairment (Aleyasin et al., 2007; Peng et al., 2019), suggesting that DJ1 may play beneficial roles to prevent neurodegeneration.

Together, these studies highlight the contribution of astrocytes to the reduction of ROS and RNS during the acute phase of ischemic stroke and the importance of astrocytic antioxidant mechanisms, such as the Nrf2-mediated pathway, as a potential therapeutic target for this early phase.

### 2.2 Excitotoxicity

Glutamate release and extracellular accumulation early after ischemia leads to neuronal damage due to overactivation of glutamate receptors (Arundine and Tymianski, 2003; Dong et al., 2009; Guo and Ma, 2021), a phenomenon known as excitotoxicity. Increased intracellular concentration of Na^+^ and Ca^2+^ through the glutamate receptors leads to ionic imbalance, mitochondrial dysfunction, protease activation, and ROS/RNS release, ultimately triggering neuronal death (Kostandy, 2012).

Ischemia-induced glutamate release occurs by three main routes: an early Ca^2+^-dependent release related to neuronal synaptic vesicle exocytosis (Katayama et al., 1991; Lee and Kim, 2015); a second route through the reversed activation or downregulation of excitatory amino-acid transporters (EAAT1 and EAAT2 in humans, GLT1 and GLAST in rodents) (Phillis et al., 2000); and a third volley through the activation of volume-sensitive outwardly rectifying anion channels (VRACs) (Phillis et al., 1997). Glutamate release through these pathways may occur at different stages of the ischemic event.

In physiological conditions, astrocytes keep extracellular glutamate levels balanced through the activation of Na^+^-dependent glutamate transporters such as GLAST (EAAT1) and GLT1 (EAAT2) (Pajarillo et al., 2019). These transporters help clear glutamate from the synaptic cleft against its electrochemical gradient by coupling the transport to the Na^+^ concentration gradient (Pajarillo et al., 2019). However, acutely after an ischemic stroke, elevated glutamate levels combined with changes in the Na^+^ concentration trigger a reversal of the high-affinity Na^+^-dependent glutamate transporters, further contributing to the extracellular accumulation of glutamate (Longuemare and Swanson, 1995; Phillis et al., 2000). In addition, hypoxia-induced activation of the inflammatory regulator nuclear factor kappa B (NFκB) (Dallas et al., 2007; Taylor, 2008; Taylor and Cummins, 2009) and high levels of extracellular glutamate (Lehmann et al., 2009) lead to downregulation of the expression of glutamate transporters GLAST and GLT1 *in vitro* (Dallas et al., 2007), increasing the extracellular glutamate concentration. After MCAO, upregulation of GLT1 using ceftriaxone (Chu et al., 2007) or by overexpression with an AAV (Harvey et al., 2011) shows a reduction in the acute accumulation of glutamate in the injured cortex, resulting in decreased neuronal cell death, smaller injuries, and improved functional recovery.

Activation of astrocytic VRACs exacerbates glutamate excitotoxicity (Kimelberg et al., 1990; Takano et al., 2005), and the use of VRAC inhibitors in rodent models of ischemic stroke during the acute phase has shown neuroprotective effects (Feustel et al., 2004). However, available inhibitors are non-selective, and results were confounded by interference with other cellular pathways (Mongin, 2016). Recently, it was shown that knock out of the obligatory subunit of astrocytic VRACs Swell1 *in vivo* results in reduced brain damage and better neurological outcomes after tMCAO (Yang et al., 2019b), further suggesting that blocking astrocytic VRAC function in the early stages of ischemic stroke may reduce glutamate-related neurodegeneration.

### 2.3 Metabolic imbalance

Astrocytes are known to provide metabolic support to neurons, but both cell types, neurons and astrocytes, possess distinct metabolic profiles. While neurons are predominantly oxidative, yielding ATP through mitochondrial activity, astrocytes are mostly glycolytic, producing pyruvate and lactate (Magistretti and Allaman, 2015; Sifat et al., 2022). This difference makes neurons more vulnerable to hypoxia and glucose restriction, as seen in models of astrocytoma (Turner and Adamson, 2011), and grants protection to astrocytes from acute cell death (Gurer et al., 2009).

Astrocyte-derived lactate can be shuttled to nearby neurons as a precursor for the tricarboxylic acid cycle (TCA) and ATP production in a process known as astrocyte-neuron lactate shuttle (ANLS) (Pellerin and Magistretti, 1994). The ANLS model propose a metabolic compartmentalization in physiological conditions with predominance of glycolysis in astrocytes and oxidative metabolism of lactate in neurons (Magistretti and Allaman, 2015). Recent work has challenged this model, suggesting that neuronal activation may not strictly depend on the ANLS as previously reported (Patel et al., 2014; Dienel, 2017). Direct uptake and oxidation of glucose in synaptosomes is substantial under both resting and bicuculline-induced activation (Patel et al., 2014), suggesting that glucose oxidation, and not astrocyte-derived lactate, plays a major role in neuronal energy production in physiological conditions. However, glucose availability decreases during ischemia, forcing neurons to use other fueling options.

Lactate production in astrocytes is enhanced under ischemic conditions, mainly through glycogen breakdown (Brown and Ransom, 2015). Lactate accumulation during the ischemic event can fuel neuronal oxidative metabolism immediately after re-oxygenation/reperfusion (Schurr et al., 2001), preventing further neuronal cell death. Lactate can then be transported from astrocytes to neurons by the monocarboxylate transporters (MCTs) (Pierre and Pellerin, 2005). MCTs subtypes present different lactate affinities and are differentially expressed in astrocytes and neurons (Pierre and Pellerin, 2005). After ischemia, cell-specific MCTs expression patterns change. MCT4, primarily expressed by astrocytes, increases within 1 h from stroke onset and returns to pre-ischemic levels within 24 h (Rosafio et al., 2016). Similarly, MCT2, enriched in neurons, increases immediately after stroke. Interestingly, neurons also upregulate MCT1 and MCT4, suggesting a neuronal metabolic reprogramming triggered by ischemia, allowing neurons to maximize their lactate usage in the acute phase and potential facilitate neuronal survival (Rosafio et al., 2016).

Under extreme situations where glucose availability is impaired, like during starvation or insulin resistance, adult neurons may rely on ketone bodies (KB) for ATP production (Takahashi, 2020). KB can enter the TCA directly in the absence of the pyruvate dehydrogenase enzyme complex (Veech, 2004). KB synthesis in astrocytes is enhanced after OGD (Takahashi et al., 2014), and therapeutic induction of KB after focal ischemia induced by MCAO results in decreased infarct area (Puchowicz et al., 2008).

Moreover, after stroke, astrocytes receive damaged mitochondria from neurons for disposal and recycling (Davis et al., 2014) and they transfer healthy mitochondria in return (Hayakawa et al., 2016). ATP levels in cortical neurons *in vitro* drop soon after OGD but can be rescued by astrocyte-derived conditioned media containing extracellular mitochondrial particles (Hayakawa et al., 2016). *In vivo*, after tMCAO, extracellular mitochondrial particles directly injected into the peri-infarct area are uptaken by neurons (Hayakawa et al., 2016). These neurons show upregulation of cell-survival-related signals, such as phospho-AKT and BCL-XL, further confirming the role of astrocyte-derived mitochondria on neuronal survival after cerebral ischemia.

Astrocytic metabolic support during ischemia can improve neuronal survival by providing metabolic precursors, like lactate and KB, and by helping maintain healthy mitochondria.

### 2.4 Edema

Brain edema is a devastating stroke complication associated with prolonged hospitalization and poor outcomes (Battey et al., 2014; Kimberly et al., 2018; McKeown et al., 2022). It consists of cerebral swelling that increases intracranial pressure, often leading to worse clinical outcomes (Leinonen et al., 2017).

The progress of cerebral edema has been divided into three stages. The cytotoxic phase, also known as cytotoxic edema, is characterized by early sudden changes to the ionic gradient that trigger cell swelling and ultimately cell death. A subsequent ionic phase promotes accumulation of interstitial fluid in the extracellular compartment before the BBB rupture. Finally, the vasogenic phase, also known as vasogenic edema, involves BBB rupture that leads to extravasation of water and plasma proteins to the interstitial space (Chen et al., 2021; Gu et al., 2022).

Astrocytes play a critical role in the generation and clearance of edema. Within minutes of the ischemic event, failure of the Na^+^/K^+^-ATPase pump leads to changes in the osmotic gradient, promoting the influx of osmotically active molecules, like Na^+^ and Cl^−^, and water to the cells triggering cell swelling in neurons, astrocytes and vascular cells (Garcia et al., 1993; O'Donnell, 2014; Rungta et al., 2015). Astrocytes are more susceptible to cell swelling due to their expression of ion transporters and water channels (Kintner et al., 2004; Risher et al., 2009). OGD-induced activation of the Na^+^-H^+^ antiporter (NHE) family member, NHE1, contributes to astrocyte swelling (Kintner et al., 2004). Astrocyte-specific NHE1 ablation in mice shows less edema, improved BBB integrity and reduced astrocyte reactivity after tMCAO (Begum et al., 2018), suggesting that astrocyte survival impedes edema progression.

Astrocyte endfeet express aquaporin 4 (AQP4), a water channel that regulates bidirectional water transport according to the osmotic gradient (Clement et al., 2020). AQP4 trafficking to the cell surface is increased in response to hypoxia-induced swelling in a calmodulin-dependent manner (Kitchen et al., 2015; Kitchen et al., 2020). Glutamate can increase astrocyte water permeability (Gunnarson et al., 2008) through the activation of the AQP4-mGluR5 complex in the astrocytic plasma membrane (Illarionova et al., 2010), leading to increase astrocytic swelling *in vitro* (Shi et al., 2017). This suggests that glutamate release by spreading depolarization after the injury could also contribute to edema formation. Ischemia also induces *de novo* upregulation of the SUR1-TRPM4 complex, a Na^+^-permeable channel that mediates monovalent cations influx in astrocytes, neurons and capillaries (Simard et al., 2006). Recent work showed that AQP4 co-assembles with SUR1-TRPM4 forming a new water/ion channel complex further contributing to astrocytic swelling (Stokum et al., 2018).

Initial studies show that AQP4 ablation reduces cerebral edema 24 h after a permanent ischemic injury (Manley et al., 2000), suggesting that AQP4 is involved in cytogenic edema and its inhibition could be a therapeutic target. However, longer time course analyses after a cerebral ischemia/reperfusion model show that AQP4 elimination leads to increased infarct volume and worse neurological scores (Zeng et al., 2012). This suggests that elimination of AQP4 may resolve initial edema but ablation of AQP4 may be detrimental in the long term. Analysis of the protein levels of AQP4 after ischemia shows a biphasic pattern with one peak happening acutely after the injury and a second increase occurring during the sub-acute phase (Ribeiro Mde et al., 2006; Zeng et al., 2012). This suggests that AQP4 could be involved in the progression of both cytotoxic and vasogenic edema, where its acute increase aids the progression of cytogenic edema, and its sub-acute increase may resolve the vasogenic edema. Timed inhibition/activation of AQP4 could provide more insights about the time-dependent role of AQP4 in edema progression and it represents a therapeutic strategy to limit edema-related injury (Clement et al., 2020).

### 2.5 Scar-border formation

In response to ischemia, astrocytes undergo a series of transcriptomic, morphological, biochemical, metabolic, and physiological changes often referred to as reactive astrogliosis (Burda and Sofroniew, 2014; Escartin et al., 2019). Astrogliosis is a continuum of changes that depend on the proximity to the injury and the triggering stimuli, ranging from subtle gene expression alterations to cell hypertrophy and scar-border formation (Anderson et al., 2014; Hart and Karimi-Abdolrezaee, 2021). Astrogliosis is triggered by many exogenous stimuli, such as inflammatory cytokines, mediators of innate immunity, hypoxia, neurotransmitters and ROS/RNS (Sofroniew, 2009; Shen et al., 2021).

Between the end of the acute phase and early stages of the sub-acute phase, astrocytes in the peri-infarct area proliferate and migrate towards the infarct border, adopting a stellate and hypertrophic morphology (Wanner et al., 2013). Reactive astrocytes interact with microglia/macrophages and initiate the formation of the scar-border via secretion of extracellular matrix proteins, such as laminin, fibronectin and chondroitin sulfate proteoglycans (CSPGs) (Sofroniew, 2009; Dias et al., 2021). The scar-border surrounds and isolates the injured tissue from the healthy brain and has traditionally been considered a physical barrier that hinders neurite outgrowth and axonal regeneration (Silver and Miller, 2004; Overman et al., 2012; Silver et al., 2014). Axonal growth inhibitory molecules such as CSPGs and ephrins are upregulated by reactive astrocytes (Silver and Miller, 2004; Overman et al., 2012). Additionally, reactive astrocytes express a broad range of inhibitory molecules, such as proinflammatory cytokines, chemokines and metalloproteinases (MMPs), which recruit peripheral leukocytes (Gliem et al., 2015) and further damage the BBB (Yang et al., 2019a) triggering neuronal cell death (Tuo et al., 2022). Therefore, preventing the formation of the scar-border was proposed to improve neuronal survival, axonal regeneration and functional recovery. However, additional studies that removed dividing reactive astrocytes in transgenic mice led to inflammation, increase in injury size, severe demyelination, reduced axonal growth and neuronal loss (Bush et al., 1999; Faulkner et al., 2004; Anderson et al., 2016), proving that the scar-border plays a major role preventing the spread of tissue damage.

Recent studies suggest that the scar-border is not an impermeable barrier. Electron microscopy scans of the reactive astrocytes within the scar-border show absence of gap junctions between astrocytes, and direct injections of dextran of different molecular weights into the injury core show dextran leakage from the core into the peri-infarct region (Zbesko et al., 2018), suggesting that molecules cross the scar-border. Moreover, isolation of extracellular fluid from the degenerating core proves to be neurotoxic *in vitro* (Zbesko et al., 2018). Increased neurodegeneration is detected in areas adjacent to the injury after pMCAO (Zbesko et al., 2018), suggesting that neurotoxic factors permeating through the scar-border contribute to the neuronal loss observed in the adjacent parenchyma. Additionally, antibodies produced by plasma cells within the injury core accumulate in the parenchyma across the scar-border (Doyle et al., 2015; Zbesko et al., 2018) and this phenomenon is linked to the development of cognitive impairment after stroke (Doyle et al., 2015).

In conclusion, the formation of the scar-border after cerebral ischemia plays a dual role. On one hand, it partially contains the spread of tissue damage. On the other hand, reactive astrocytes within the scar-border may exacerbate neural tissue damage and prevent axonal growth. Modulation of specific subpopulations of reactive astrocytes may help reduce their harmful effects without compromising their beneficial functions.

### 2.6 Neuroinflammation

In the context of cerebral ischemia, it has been proposed that microglia react first to many injury signals, such as danger-associated molecular patterns (DAMPs), leading to cytokine release (IL6, IL1A and TNFα) and further reactive astrogliosis (Sofroniew, 2014; Liddelow and Barres, 2017). In turn, reactive astrocytes aggravate the inflammatory response by releasing a large number of inflammatory factors, such as cytokines, chemokines, ATP and ROS/RNS, all affecting neuronal viability (Linnerbauer et al., 2020; Li et al., 2022a).

Transcriptomic analyses show that astrocytes exposed to different physiopathological contexts can express specific subsets of genes (Zamanian et al., 2012; Liddelow et al., 2017; Matusova et al., 2023). For example, subsets of reactive astrocytes induced by tMCAO adopt a neuroprotective phenotype characterized by upregulation of genes related with cell survival and synapse formation (Zamanian et al., 2012; Rakers et al., 2019). Subpopulations of reactive astrocytes triggered by IL1a, TNFα and C1q secreted by microglia lose their ability to induce synapse formation, reduce phagocytosis of synaptosomes and myelin debris, and increase neurotoxicity (Liddelow et al., 2017). These studies highlight the heterogeneity of reactive astrocytes and open the possibility for therapeutic targeting of specific subsets of reactive astrocytes.

Peripheral immune cells, such as neutrophils, monocytes and T cells, to enter the CNS parenchyma upon BBB breakdown and modulate neuroinflammation (Koyama and Shichita, 2023). IL15 is a proinflammatory cytokine involved in regulating the magnitude of the immune response by promoting the migration of T cells to inflammatory tissue and maintaining homeostasis and cytotoxic activity in lymphocytes (Zhu et al., 2022b). After stroke, increased production of IL15 by astrocytes is necessary and sufficient to promote activation and accumulation of CD8+T and NK cells in the ischemic brain (Li et al., 2017). Astrocyte-specific overexpression of IL15 leads to bigger infarct area and worse motor function after MCAO (Li et al., 2017). On the contrary, neutralizing IL15 decreases the effector capacity of NK, CD8+T and CD4+T cells after MCAO, and ablation of this cytokine leads to reduced brain infarction and improved neurological function (Lee et al., 2018). Together, these studies shed light on the contribution of astrocytes to lymphocyte activation and accumulation in the injured tissue, positioning astrocytic IL15 as a therapeutic target to restrict the inflammatory response that leads to neuronal cytotoxicity.

Astrocytes also exert an anti-inflammatory role by secreting anti-inflammatory and neurotrophic factors (Colombo and Farina, 2016; Linnerbauer and Rothhammer, 2020). Transforming growth factor beta (TGFβ) is a key immune system modulator with neuroprotective effects (Buisson et al., 2003; Luo, 2022), highly upregulated in microglia/macrophages and astrocytes after stroke (Doyle et al., 2010). Specific inhibition of TGFβ signaling in astrocytes leads to a significant increase in immune cell infiltration and reactivity in the peri-infarct area 3 days after ischemia (Cekanaviciute et al., 2014). Transgenic mice where TGFβ signaling is obliterated show increased neuroinflammation and in consequence, worse neurological function as well as delayed injury expansion in a model of photothrombotic stroke (Cekanaviciute et al., 2014). This demonstrates that activation of astrocytic TGFβ signaling is a mechanism of neuroinflammation resolution with potential to prevent neurodegeneration at late stages.

Neuroinflammation is a key driver for neurodegeneration after stroke, and it is heavily modulated by the interplay between astrocytes and other inflammatory cells (Jayaraj et al., 2019). Reactive astrocytes play a dual role in the neuroinflammatory response, and a deeper understanding of the astrocyte subpopulations would aid to design future successful therapeutic approaches.

## 3 Astrocytes during the sub-acute phase

### 3.1 Neurogenesis and astrogliogenesis

Neurogenesis has been observed throughout adulthood in distinct regions of the brain, specifically in the subventricular zone (SVZ) located adjacent to the lateral ventricle and the subgranular layer (SGL) of the dentate gyrus (DG) in the hippocampus (Tobin et al., 2014).

After focal ischemia, there is increased proliferation of neuroblasts in the SVZ (Arvidsson et al., 2002; Parent et al., 2002). These neuroblasts then migrate from the SVZ into the peri-infarct area, further differentiating into mature neurons (Arvidsson et al., 2002; Parent et al., 2002). Astrocytes have been implicated in triggering and guiding this neurogenic response. Astrocytic Ca^2+^ waves transmitted through gap junctions in the SVZ enhanced the self-renewal and migratory capacity of neural stem/progenitor cells (NSPCs) via activation of Notch signaling pathway as seen in organotypic brain slices of a mouse stroke model (Kraft et al., 2017). Similarly, after OGD, the astrocyte-derived DAMP high-mobility group box 1 (HMGB1) increased NSPCs proliferation through activation of the PI3K/Akt signaling pathway (Li et al., 2014). Reactive astrocytes in the peri-infarct area upregulate the chemokine stromal cell-derived factor 1a (SDF1a), which binds to its receptor CXCR4 expressed in NSPCs (Imitola et al., 2004). This suggests that astrocytic SDF1a acts as a homing molecule for the NSPCs of the SVZ after an ischemic stroke. These results highlight the role of astrocytes in neuroblast migration and differentiation into mature neurons in the peri-infarct.

Neurogenesis triggered by ischemic stroke has been demonstrated in animal models (Ceanga et al., 2021) and human post-mortem tissue (Passarelli et al., 2022). These newly formed neurons migrate and integrate into the local circuitry, form synapses and firing action potentials (Ceanga et al., 2021). However, there is no direct evidence that they contribute to functional recovery. Survival of these newly formed neurons is very poor, estimating that approximately 80% of the new neurons die within 2 weeks post-stroke (Arvidsson et al., 2002; Marques et al., 2019). The role of astrocytes in the survival of these newly formed neurons has yet to be established.

Astrogliogenesis may also occur from SVZ NSPCs, and these newly generated astrocytes migrate to the injury site and contribute to scar-border formation (Benner et al., 2013; Faiz et al., 2015; Pous et al., 2020). SVZ-derived astrocytes express high levels of thrombospondin 4 (TSP4), and SVZ astrogliogenesis is reduced in the TSP4 KO mice after photothrombosis (Benner et al., 2013). Neurogenesis is increased in the TSP4 KO mouse along with abnormal scar-border formation and a significant increase of microvascular hemorrhages in the peri-infarct area (Benner et al., 2013), suggesting that suppression of astrogliogenesis in the SVZ after ischemic stroke could hinder recovery. Recent work has shown that blood-derived fibrinogen promotes NSPCs differentiation into astrocytes through the activation of the bone morphogenetic protein (BMP) receptor signaling pathway (Pous et al., 2020). Pharmacological depletion of fibrinogen before focal ischemia leads to a reduced number of TSP4+ astrocytes in the peri-infarct area (Pous et al., 2020), suggesting that exogenous factors may directly regulate the differentiation of adult NSPCs into astrocytes. Astrogliogenesis from the SVZ contributes to the scar-border formation, but the molecular signature of these newly formed astrocytes seems to differ from the parenchymal reactive astrocytes (Benner et al., 2013). The factors that trigger astrogliogenesis over neurogenesis in the SVZ or whether these SVZ-derived astrocytes contribute differently to neuronal survival in the peri-infarct area is yet to be defined.

Parenchymal astrocytes are endogenously reprogrammed into neuronal cells after cerebral ischemia, regulated by Notch1 signaling pathways (Shimada et al., 2012; Magnusson et al., 2014). Some studies show that pharmacological blockade of Notch1 signaling *in vivo* successfully promote astrocyte transdifferentiation (Shimada et al., 2012; Hao et al., 2023). Cell fate-mapping analyses of astrocytes show that after injury some astrocytes acquire stem cell properties which may represent a source for neuronal repopulation (Buffo et al., 2008). These astrocyte-derived new neurons form synapses and fire action potentials (Duan et al., 2015), suggesting that promoting astrocyte transdifferentiation during the sub-acute phase may counteract the neuronal loss that occurred at earlier post-stroke phases, with potential benefits to limit neurodegeneration.

A recent study showed that endothelial cells might also trigger astrocyte transdifferentiation after stroke by a non-cell autonomous mechanism (Li et al., 2022b). After OGD, brain endothelial cells release microvesicles containing the pro-neural transcription factor ASCL1 that triggers astrocyte transdifferentiation. Similarly, after focal ischemia *in vivo*, endothelial cells upregulate ASCL1 prior to local astrocytic conversion to NSCs, and overexpression of ASCL1 in endothelial cells correlates with improved motor function (Li et al., 2022b).

Neurogenesis and astrogliogenesis have important implications in recovery from ischemic injuries, and understanding the contribution of astrocytes to these processes may reveal new targets to develop future therapeutic strategies.

### 3.2 Synaptic plasticity

After an ischemic stroke, there is a reduction in dendritic spine density in the peri-infarct area (Brown et al., 2008; Mostany et al., 2010), followed by restoration of spines over time (Brown et al., 2007). This gain of dendritic spines in the peri-infarct area correlates with functional recovery observed during the sub-acute phase (Brown et al., 2009), suggesting that dendritic spine turnover provides a substrate for neurological improvement.

Astrocytes detect and respond to neuronal activity, influencing the formation of synapses during development and adulthood (Allen and Eroglu, 2017). Several factors regulating neuronal synapse formation and maturation have been identified, including thrombospondins (TSP), hevin, SPARC, glypicans and Chrdl1 (Christopherson et al., 2005; Kucukdereli et al., 2011; Allen et al., 2012; Blanco-Suarez et al., 2018). These astrocyte-secreted factors and their roles in synaptic plasticity may have important implications in stroke-induced synaptic loss and neurodegeneration, although just a few have been studied in this context, showing injury-induced upregulation during the sub-acute phase.

Thrombospondins (TSPs) are large extracellular matrix proteins mainly involved in platelet aggregation, inflammation, angiogenesis, and synaptogenesis (Adams and Lawler, 2011; Wang et al., 2012). After cerebral ischemia, thrombospondin 2 (TSP2) is highly upregulated during the sub-acute phase, while thrombospondin 1 (TSP1) shows a more transient and earlier upregulation (Liauw et al., 2008). Eliminating both TSP1/2 restricts recovery by limiting synaptic restoration and axonal sprouting, which translates into impaired motor function (Liauw et al., 2008).

Hevin, another astrocyte-secreted protein, modulates the formation of glutamatergic synapses in the developing brain (Kucukdereli et al., 2011; Singh et al., 2016; Gan and Sudhof, 2020). Hevin expression is increased in the peri-infarct reactive astrocytes after a focal ischemic injury in both the striatum (Lively et al., 2011) and the motor cortex (Kim et al., 2021). Hevin interacts with the neuron-specific vesicular protein calcyon in both physiological and pathological contexts (Kim et al., 2021). Ablation of either hevin or calcyon exacerbates synaptic loss and hinders functional recovery, as seen in a photothrombotic mouse model of focal ischemia (Kim et al., 2021).

SPARC is highly expressed by astrocytes and microglia during development but reduced in adult astrocytes (Vincent et al., 2008; Kucukdereli et al., 2011). In physiological conditions SPARC antagonizes the effects of hevin (Kucukdereli et al., 2011) by inhibiting postsynaptic B3 integrin and destabilizing AMPA receptors (AMPARs) in the postsynaptic membrane (Jones et al., 2011). In response to ischemic conditions, both *in vitro* and *in vivo,* SPARC is upregulated in reactive astrocytes and microglia, and it is attributed a neuroprotective role (Lloyd-Burton et al., 2013; Jones et al., 2018). However, how the SPARC interaction with hevin is affected during ischemic stroke is not known and further experiments are needed to fully understand the neuroprotective role of SPARC.

Chrdl1 promotes synaptic maturation by recruiting Ca^2+^-impermeable GluA2-containing AMPARs to synaptic sites and limiting experience-dependent plasticity in the cortex (Blanco-Suarez et al., 2018). In a photothrombotic model of ischemic stroke, Chrdl1 is upregulated in the peri-infarct astrocytes. Absence of Chrdl1 in a global KO mouse model prevents ischemia-driven dendritic spine loss in the peri-infarct area and limits apoptotic cell death (Blanco-Suarez and Allen, 2022), potentially providing a favorable environment for neuronal survival.

Astrocytes can also phagocytose unnecessary excitatory synapses in the hippocampus of the adult mouse brain, helping maintain circuit homeostasis (Lee et al., 2021). Transcriptomic analysis show that astrocytes are enriched in genes involved in engulfment pathways in both development (Cahoy et al., 2008) and after stroke (Rakers et al., 2019). In response to stroke, reactive astrocytes engulf synapses through MEGF10, MERTK and GULP1-related pathways (Morizawa et al., 2017; Shi et al., 2021). Reactive astrocytes in the peri-infarct area upregulate phagocytic markers at 7–14 days post-stroke, suggesting that astrocyte phagocytic function is related to plastic changes occurring during the sub-acute phase (Morizawa et al., 2017; Shi et al., 2021). Inducible ablation of astrocytic MEGF10 or MERTK reduces synaptic engulfment in the peri-infarct region and increases the number of dendritic spines 14 days after stroke, which also promotes enhanced functional recovery (Shi et al., 2021). This suggests that preventing astrocyte-driven synaptic engulfment in the peri-infarct area favors dendritic spine turnover and improves behavioral outcomes.

### 3.3 Axonal sprouting

In both human and animal models, spontaneous recovery of sensory-motor function after ischemic stroke is linked to axonal sprouting of different axonal tracts (Carmichael et al., 2017). Axonal sprouting occurs in areas adjacent to the infarct as well as in areas functionally connected but physically distant from the lesion, such as the contralateral hemisphere and the spinal cord (Carmichael et al., 2017). This remodeling can be detected as early as 1 week after stroke (Li et al., 2010) and extend into several months after the ischemic injury (Dancause et al., 2005; Brown et al., 2009), suggesting that the molecular changes triggering axonal growth occur early after ischemia and permanently impact the post-stroke neural architecture.

Among the many molecules that modulate axonal growth and trigger axonal sprouting, astrocyte-related molecules have gained interest in the past decade (Benowitz and Carmichael, 2010). Reactive astrocytes produce molecules that block axonal sprouting, such as chondroitin sulfate proteoglycans (CGSPs) and Ephrin A5 (Deguchi et al., 2005; Overman et al., 2012). CSPGs have been extensively studied as axonal growth inhibitors during development and after injury of the CNS (Galtrey and Fawcett, 2007). Perilesional CSPG digestion with chondroitinase (Gherardini et al., 2015) or ablation of the transcription factor modulating the expression of astrocytic CSPGs (Sox 9) (Xu et al., 2018) led to increased neuroplasticity that correlates with improved forelimb motor function after cerebral ischemia. Similarly, Ephrin A5, a development-associated growth inhibitor, is upregulated in astrocytes after ischemic stroke (Overman et al., 2012), and local inhibition of Ephrin A5 signaling in the sub-acute phase promotes axonal growth and functional recovery (Overman et al., 2012). Blocking reactive astrocyte-derived molecules can benefit functional recovery after stroke, given their role in promoting axonal regeneration.

However, attenuating astrocyte reactivity by double KO of GFAP and vimentin has shown detrimental effects. Motor function and distant axonal sprouting in the cervical spinal cord were significantly reduced in the double KO mice after a focal ischemic injury (Liu et al., 2014). These results were recently confirmed by resting-state functional magnetic resonance imaging (rs-fMRI), where a less stable functional network in the double KO mice in the sub-acute phase suggested maladaptive axonal plasticity after stroke (Aswendt et al., 2022). Interestingly, overexpression of leucine zipper-bearing kinase (LZK), a positive modulator of astrogliosis, resulted in increased distant axonal sprouting after stroke in the cervical region and increased production of ciliary neurotrophic factor (CNTF) by the surrounding astrocytes (Chen et al., 2022). These results point out that astrocyte heterogeneity, microenvironmental cues and distance from the injury may influence the contribution of astrocyte reactivity to axonal growth.

Other astrocyte-secreted molecules have been linked to collateral sprouting. TSP1 and 2 are upregulated in the peri-infarct astrocytes, and their elimination leads to significant decrease in axonal sprouting, and it impairs functional recovery (Liauw et al., 2008). However, the mechanisms underlying this TSP1 and TSP2 effect are not elucidated.

Astrocyte modulation of the local environment in the parenchyma, both proximal and distal from the ischemic lesion, contributes to axonal sprouting and, ultimately, spontaneous functional recovery. Whether these changes are specific to astrocyte subpopulations near or far from the area affected by stroke is yet to be described.

### 3.4 Angiogenesis and vascular repair

Angiogenesis and blood-brain barrier (BBB) repair are crucial for stroke recovery. Angiogenesis is promptly induced after stroke, however, many new microvessels are leaky and do not last long (Yu et al., 2007). The BBB can remain disrupted for up to 2 weeks (Abulrob et al., 2008), contributing to vasogenic edema and immune cell infiltration (Stokum et al., 2015). Astrocytes are involved in these post-stroke processes via the following mechanisms.

Recent work using *in vivo* two-photon imaging shows that astrocytes contact newly formed vessels in the peri-infarct area (Williamson et al., 2021). Chemogenetic ablation of reactive astrocytes after stroke shows impaired vascular remodeling, increased vascular permeability, and cell death in the peri-infarct area during the sub-acute phase, along with worse functional recovery (Williamson et al., 2021). This highlights the importance of astrocyte-vessel interactions to prevent neurodegeneration.

Sonic hedgehog (Shh), a glycoprotein primarily studied in patterning of the CNS during development, has been linked to this astrocyte-mediated repair after stroke (Hill et al., 2021). Shh is secreted *in vitro* by astrocytes exposed to OGD and induces proliferation, migration and tube formation of brain microvascular endothelial cells through the RhoA/ROCK pathway (He et al., 2013). Exogenous delivery of Shh into the peri-infarct area after MCAO decreases brain water content and BBB permeability and increases expression of tight junction proteins ZO-1 and occludin (Xia et al., 2013), suggesting that astrocyte-derived Shh mediates BBB repair and promotes angiogenesis with important implications in restricting vascular damage and subsequent neurodegeneration.

## 4 Astrocytes in the chronic phase

### 4.1 Post-stroke cognitive impairment

Stroke increases the risk of cognitive impairment (Rost et al., 2022), representing a daunting long-term consequence for stroke survivors (McKevitt et al., 2011; Pollock et al., 2012). Despite the important burden that this represents, preclinical studies to underpin the mechanisms that lead to post-stroke cognitive decline are scarce.

Post-stroke cognitive impairment (PSCI) is classified as a major subtype of vascular cognitive impairment (VCI) (Mijajlovic et al., 2017). Patients with PSCI may have damage in one or more cognitive domains, but the most prevalent problems are memory, visuoconstructional and spatial function and executive function and attention (Jokinen et al., 2015). Various risk factors and underlying conditions have been linked to the development of PSCI, such as genetic predispositions, pre-stroke cognitive status, previous history of stroke, vascular risk factors and co-morbidities (Hu and Chen, 2017; Mijajlovic et al., 2017). Several mechanisms have been proposed to contribute to the cognitive deficit of cerebrovascular lesions, such as changes in blood and oxygen supply, chronic inflammation, disruption of the glymphatic clearance, disruption of axonal tracts and altered connectivity (Iadecola, 2010; Hu and Chen, 2017).

Focal ischemic stroke triggers a wave of secondary damage affecting distant brain areas anatomically connected to the injured tissue but unaffected by the initial lesion. This is known as secondary neurodegeneration and it is characterized by neuronal loss, neuroinflammation and accumulation of neurotoxic proteins (Zhang et al., 2012; Stuckey et al., 2021). After cortical focal ischemia, an increase in neuronal atrophy, reactive microglia, and reactive astrocytes in the hippocampus is associated with worsened spatial learning and memory in mice (Schmidt et al., 2015; Sanchez-Bezanilla et al., 2021). Genetic ablation of the astrocytic receptor P2Y1 involved in proinflammatory cytokine release prevents cognitive impairments after MCAO but has no effects on sensorimotor decline (Chin et al., 2013). This correlates with decrease astrocyte reactivity and neuroinflammation in the hippocampus, suggesting that neuroinflammation triggered by secondary neurodegeneration plays a role in the development of PSCI (Chin et al., 2013). Similarly, environmental enrichment after MCAO improves spatial memory along with brain-derived neurotrophic factor (BDNF) production in the hippocampus and reduction of oxidative stress markers (Zhang et al., 2021), suggesting that oxidative stress may be a factor that exacerbates cognitive impairments after ischemic lesions.

Focal ischemia followed by chronic hypoperfusion triggers changes in AQP4 distribution from perivascular to parenchymal and increases amyloid-β deposits which contributes to PSCI (Back et al., 2017). AQP4 is part of the glymphatic system, a paravascular pathway that helps solute clearance from the interstitial space (Iliff et al., 2012), suggesting that alterations in this protein may affect the clearance of neurotoxic metabolites contributing to the exacerbation of cognitive deficits at later post-stroke stages.

Permeability of the scar-border allows the accumulation of antibodies in the parenchyma after stroke (Doyle et al., 2015; Zbesko et al., 2018), which is linked to the development of PSCI (Doyle et al., 2015; Doyle and Buckwalter, 2017). B-cell infiltration to the injury core correlates with a progressive deficit in long-term potentiation (LTP) and spatial memory dysfunction after pMCAO (Doyle et al., 2015). This cognitive deficit is absent in animals lacking mature B cells, further suggesting that B cell infiltration plays a crucial role in PSCI development in mice (Doyle et al., 2015). Whether changes to the scar-border permeability help prevent or delay the development of PSCI is still unclear.

Neuroinflammation and oxidative stress linked to secondary neurodegeneration after ischemic stroke is proposed to drive the development of PSCI. In addition, disruption of neurotoxic metabolite clearance due to changes in the glymphatic system also appears to contribute to PSCI development. Astrocytes play a major role in secondary neurodegeneration and neurotoxic metabolite clearance, and further dissection of their specific contribution in the chronic phase will help guide future therapeutic interventions at late post-stroke stages when we currently lack effective approaches.

## 5 Discussion

During the acute phase after ischemic stroke, astrocytes modulate oxidative stress, metabolic support, form the scar-border, regulate the development of cytotoxic and vasogenic edema and contribute to neuroinflammation, giving them a range of roles that may result in beneficial and detrimental effects. During the sub-acute phase, astrocytes help modulate neurogenesis and astrogliogenesis, synaptic plasticity, axonal growth, BBB integrity and angiogenesis, paving the road for neuronal survival and spontaneous motor and sensory recovery. During the chronic phase, cognitive impairment due to secondary neurodegeneration appears; however, astrocyte contributions to this pathology are not entirely understood.

Astrocytes play crucial roles in brain homeostasis, and they are major determinants of stroke outcomes. Due to their heterogeneity, astrocytes play a plethora of roles in stroke progression, which makes it impossible to simply classify them as “good” or “bad.” Research on astrocyte biology has gained momentum in recent years, and several astrocytic molecular targets with therapeutic potential have been identified in the context of ischemic stroke (Table 1). However, many open questions remain to understand the complex role of astrocytes under different pathological conditions. As we emphasized throughout this review, astrocytes complex responses contribute to positive and negative outcomes in the context of ischemic stroke. Which factors determine whether astrocytes adopt a beneficial or detrimental role? Is the response of reactive astrocyte subpopulations depending on the same factors in other disease conditions? For example, reactive astrocytes differ in their response depending on the timepoint considered from onset of the ischemic insult, or their proximity to the core of the ischemic injury. This highlights the morphological, functional, and spatiotemporal heterogeneity of astrocytes, and strongly suggests that accounting for the affected CNS region, pathological stimuli, and/or timepoint after initial stimulus will dictate the astrocytic response. These are important considerations when designing new pre-clinical studies or extrapolating to different pathological conditions. Current research is looking into astrocyte heterogeneity and their potential implications in the progression of chronic conditions such as Parkinson’s and Alzheimer’s disease (Ramos-Gonzalez et al., 2021).

**TABLE 1:** Astrocytic targets identified in the post-ischemic brain in pre-clinical studies and experimental approach to prevent pathophysiological mechanisms.

Astrocytic target	Pathophysiological mechanism	Experimental approach	Species and stroke model	References
Nrf2	Oxidative stress	Pharmacological upregulation of astrocytic Nrf2	Mouse, tMCAO	Hong et al. (2023)
				Takagi et al. (2014)
DJ1	Oxidative stress	Downregulation of DJ1	Rat, tMCAO	Aleyasin et al. (2007)
			Mouse, endothelin-1	Peng et al. (2019)
GLT1	Excitotoxicity	Pharmacological or genetic upregulation of GLT1	Rat, tMCAO	Harvey et al. (2011)
				Chu et al. (2007)
VRACs	Excitotoxicity	Tamoxifen inhibition of VRACs	Rat, tMCAO	Feustel et al. (2004)
		Elimination of Swell1 in astrocytes	Mouse, tMCAO	Yang et al. (2019a)
KB	Metabolic imbalance	Diet-induced production of KBs	Rat, tMCAO	Puchowicz et al., 2008
Mitochondria	Metabolic imbalance	Infusion of exogenous mitochondrial particles into the cerebral cortex	Mouse, tMCAO	Hayakawa et al. (2016)
NHE1	Edema	Elimination of NHE1	Mouse, tMCAO	Begum et al. (2018)
AQP4	Edema	Elimination of AQP4 (only beneficial during the acute phase)	Mouse, pMCAO	Zeng et al. (2012)
				Manley et al. (2000)
Ephrin A5	Reactive astrogliosis	Blocking of EphrinA5 by EphA5-Fc delivery via hydrogel	Mouse, photothrombosis	Overman et al. (2012)
CSPGs	Reactive astrogliosis, axonal growth	Perilesional infusion of chondroitinase to digest CSPGs	Rat, endothelin-1	Gherardini et al. (2015)
MEGF10	Neuroinflammation	Upregulation of MEGF10 and MERTK-mediated signaling	Mouse, tMCAO	Shi et al. (2021)
MERTK				Morizawa et al. (2017)
IL15	Neuroinflammation	Blocking IL15 with neutralizing antibodies or genetic elimination	Mouse, tMCAO	Li et al. (2017)
				Lee et al. (2018)
Notch1	Neurogenesis	Activation of Notch1 signaling	Mouse, pMCAO	Kraft et al. (2017)
SDF1	Neurogenesis	Increase SDF1 to promote neurogenesis	Mouse, MCAO	Imitola et al. (2004)
ASCL1	Neurogenesis	Intracortical injection of endothelial-derived microvesicles to deliver ASCL1	Mouse, tMCAO	Li et al. (2022b)
Chrdl1	Synaptic plasticity	Elimination of Chrdl1	Mouse, photothrombosis	Blanco-Suarez and Allen (2022)
LZK	Axonal growth	Astrocyte-specific overexpression of LZK.	Mouse, photothrombosis	Chen et al. (2022)
Shh	BBB disruption	Intraventricular injection of Shh	Rat, pMCAO	Xia et al. (2013)
P2Y1	PSCI	Elimination of P2Y1	Mouse, tMCAO	Chin et al. (2013)

Thus, closer evaluation of the factors (time, location, stimuli) that determine the astrocytic function and their complex responses and interactions with other CNS cell types under diverse pathological conditions is necessary to unlock new approaches to design astrocyte-targeting therapies. Further studies are needed to fully assess the potential therapeutic application and translation to the clinic of astrocyte-based interventions to mitigate neurodegeneration in both acute and chronic conditions.

## Figures and Tables

**Table:** 

**AAV**	Adeno-associated virus
**AMPAR**	α-amino-3-hydroxy-5-methyl-4-isoxazolepropionic acid receptor
**ANLS**	Astrocyte-neuron lactate shuttle
**AQP4**	Aquaporin 4
**ATP**	Adenosine triphosphate
**BBB**	Blood-brain barrier
**BDNF**	Brain-derived neurotrophic factor
**BMP**	Bone morphogenetic protein
**CAT**	Catalase
**CCA**	Common carotid artery
**Chrdl1**	Chordin-like 1
**CNTF**	Ciliary neurotrophic factor
**CSF**	Cerebrospinal fluid
**CSPG**	Chondroitin sulfate proteoglycan
**DAMPs**	Danger-associated molecular pattern
**DG**	Dentate gyrus
**EAAT**	Excitatory amino-acid transporter
**ECA**	External carotid artery
**ET-1**	Endothelin-1
**GFAP**	Glial fibrillary acidic protein
**GLAST**	Glutamate aspartate transporter
**GLT1**	Glutamate transporter 1
**GSH**	Glutathione
**HMGB1**	High-mobility group box 1
**ICA**	Internal carotid artery
**IL6**	Interleukine 6
**IL1A**	Interleukine 1A
**IL15**	Interleukine 15
**KB**	Ketone bodies
**KO**	Knockout
**LTP**	Long-term potentiation
**LZK**	Leucine zipper-bearing kinase
**MCT**	Monocarboxylate transporter
**MCA**	Middle cerebral artery
**MCAO**	Middle cerebral artery occlusion
**mGluR5**	Metabotropic glutamate receptor 5
**MMP**	Metalloproteinase
**MRI**	Magnetic resonance imaging
**NFkB**	Nuclear factor kappa B
**NHE**	Na^+^ - H^+^ antiporter
**Nrf2**	Nuclear factor erythroid-2 related factor
**NSPC**	Neural stem progenitor cell
**OGD**	Oxygen and glucose deprivation
**pMCAO**	Permanent middle cerebral artery occlusion
**PSCI**	Post-stroke cognitive impairment
**RNS**	Reactive nitrogen species
**ROS**	Reactive oxygen species
**rs-fMRI**	Resting-state functional magnetic resonance imaging
**SDF1a**	Stromal cell-derived factor 1a
**SGL**	Subgranular layer
**Shh**	Sonic hedgehog
**SPARC**	Secreted protein acidic and cysteine rich
**SOD**	Superoxide dismutase
**SUR1-TRPM4 complex**	Sulfonyl-urea receptor 1—transient receptor potential melastatin 4
**SVZ**	Subventricular zone
**TCA**	Tricarboxylic acid cycle
**TGFb**	Transforming growth factor beta
**tMCAO**	Transient middle cerebral artery occlusion
**TNF**	Tumor necrosis factor
**TSP**	Thrombospondin
**2-VO**	Two-vessel occlusion
**4-VO**	Four-vessel occlusion
**VCI**	Vascular cognitive impairment
**VRAC**	Volume-sensitive outwardly rectifying anion channels
