# Supplementary material for: Astrocytes in stroke-induced neurodegeneration: a timeline
Source: Front Mol Med. 2023 Sep 7;3:1240862. doi: 10.3389/fmmed.2023.1240862 (PMC11285566; doi:10.3389/fmmed.2023.1240862)
Supplement: Supplementary file 1 [file Table1.DOCX]

Table 1. Most common *in vitro* and *in vivo* models of stroke.

| **Model** | **Procedure** | **Description** | **Outcome** | **Advantages** | **Limitations** | **References** |
| --- | --- | --- | --- | --- | --- | --- |
| *In vitro* | Oxygen and glucose deprivation (OGD). | Cells are incubated in a glucose-free medium under deoxygenated atmosphere. | Cell viability is compromised. | Explore mechanisms and molecular pathways involved in cell death and cell-to-cell communication. | Difficult to mimic reperfusion.  Lack of infiltrating leukocytes. | (Tasca, Dal-Cim et al. 2015) |
| *In vivo* | Global ischemia by two-vessel occlusion (2-VO). | Reversible or permanent bilateral occlusion of the CCA. | Bilateral hemispheric ischemia. | Single surgery. | High mortality depending on animal strain.  High variability.  Potential development of seizures. | (Eklöf and Siesjö 1972, Chen, Chen et al. 2019) |
|  | Global ischemia by four-vessel occlusion (4-VO). | Two-day procedure. Day 1, permanent occlusion of vertebral arteries by electrocoagulation. Day2, reversible occlusion of common carotid arteries (CCA). | Bilateral hemispheric ischemia including brainstem damage. | Partially reversible. | Two-day procedure.  High mortality depending on animal strain. | (Pulsinelli and Brierley 1979, Yamaguchi, Calvert et al. 2005) |
|  | Focal ischemia by transient MCAO (tMCAO). | A filament is fed into the middle cerebral artery (MCA)from either the external (ECA) or internal carotid arteries (ICA) to block blood flow for a set amount of time | Large volume infarct affecting ipsilateral frontal cortex and lateral part of the neostriatum. | Controllable reperfusion. | High mortality rate.  Lesion size is variable and depends on animal strain.  Requires a second surgery to remove filament. | (Chiang, Messing et al. 2011, Engel, Kolodziej et al. 2011) |
|  | Focal ischemia by permanent MCAO (pMCAO) | A filament is fed into the MCA from either the ECA or ICA to block blood flow and its left there.  OR  Direct occlusion of the MCA after craniotomy by either suturing, cauterizing, transection or photothrombotic occlusion. | Large volume infarct affecting ipsilateral frontal cortex and lateral part of the neostriatum. | Single surgery. | High mortality.  Lesion size is variable and depends on animal strain.  Requires a second surgery to remove filament. | (Chiang, Messing et al. 2011, Engel, Kolodziej et al. 2011) |
|  | Focal ischemia by endothelin-1 (ET-1) occlusion. | Local application of endothelin-1 to MCA.  OR  Intracerebral stereotaxic injection on the cortical surface to induce reversible vasoconstriction with consequent reduction in blood flow. | Small infarct. | Less invasive.  Highly reproducible injury.  Low mortality rate.  Size and location of the injury is experimentally controlled. | Reperfusion is not controlled.  ET-1 has effects on neurons and astrocytes. | (Sharkey, Ritchie et al. 1993, Reid, Dawson et al. 1995, Horie, Maag et al. 2008) |
|  | Focal ischemia by photothrombosis. | Systemic injection of photoactivatable dye rose Bengal and transcranial illumination with a light source. | Small infarct. | Less invasive.  Highly reproducible injury.  Low mortality rate. Size and location of the injury is experimentally controlled. | Reperfusion is not controlled.  Small peri-infarct region. | (Watson, Dietrich et al. 1985, Labat-gest and Tomasi 2013, Clark, Sullender et al. 2019, Blanco-Suárez 2023) |
|  | Hemorrhagic stroke by intracerebral blood injection | Stereotaxic intracerebral injection of autologous blood. | Small infarct. | Mimic the hematoma and blood toxicity.  Size and location of the injury is experimentally controlled. | Does not involved major vessel rupture. | (Ropper and Zervas 1982, Deinsberger, Vogel et al. 1996, Klebe, Iniaghe et al. 2018) |
|  | Hemorrhagic stroke by collagenase injection | Stereotaxic intracerebral injection of collagenase. | Small infarct. | Spontaneous bleeding.  Controllable hematoma size.  Size and location of the injury is experimentally controlled. | Bleeding is slow and only ruptures small vessels.  Exacerbated inflammatory response. | (Klebe, Iniaghe et al. 2018, Rosenberg, Mun-Bryce et al. 2018) |

Blanco-Suárez, E. (2023). "Photothrombotic Model to Create an Infarct in the Hippocampus." Methods Mol Biol **2616**: 29-38.

Chen, C. Y., R. J. Chen and G. A. Lee (2019). "Two-vessel Occlusion Mouse Model of Cerebral Ischemia-reperfusion." J Vis Exp(145).

Chiang, T., R. O. Messing and W. H. Chou (2011). "Mouse model of middle cerebral artery occlusion." J Vis Exp(48).

Clark, T. A., C. Sullender, S. M. Kazmi, B. L. Speetles, M. R. Williamson, D. M. Palmberg, A. K. Dunn and T. A. Jones (2019). "Artery targeted photothrombosis widens the vascular penumbra, instigates peri-infarct neovascularization and models forelimb impairments." Scientific Reports **9**(1): 2323.

Deinsberger, W., J. Vogel, W. Kuschinsky, L. M. Auer and D.-K. Böker (1996). "Experimental intracerebral hemorrhage: Description of a double injection model in rats." Neurological Research **18**(5): 475-477.

Eklöf, B. and B. K. Siesjö (1972). "The Effect of Bilateral Carotid Artery Ligation upon the Blood Flow and the Energy State of the Rat Brain." Acta Physiologica Scandinavica **86**(2): 155-165.

Engel, O., S. Kolodziej, U. Dirnagl and V. Prinz (2011). "Modeling stroke in mice - middle cerebral artery occlusion with the filament model." J Vis Exp(47).

Horie, N., A.-L. Maag, S. A. Hamilton, H. Shichinohe, T. M. Bliss and G. K. Steinberg (2008). "Mouse model of focal cerebral ischemia using endothelin-1." Journal of Neuroscience Methods **173**(2): 286-290.

Klebe, D., L. Iniaghe, S. Burchell, C. Reis, O. Akyol, J. Tang and J. H. Zhang (2018). "Traumatic and Ischemic Injury, Methods and Protocols." Methods in Molecular Biology **1717**: 83-91.

Labat-gest, V. and S. Tomasi (2013). "Photothrombotic Ischemia: A Minimally Invasive and Reproducible Photochemical Cortical Lesion Model for Mouse Stroke Studies." Journal of Visualized Experiments(76).

Pulsinelli, W. A. and J. B. Brierley (1979). "A new model of bilateral hemispheric ischemia in the unanesthetized rat." Stroke **10**(3): 267-272.

Reid, J. L., D. Dawson and I. M. Macrae (1995). "Endothelin, Cerebral Ischaemia and Infarction." Clinical and Experimental Hypertension **17**(1-2): 399-407.

Ropper, A. H. and N. T. Zervas (1982). "Cerebral blood flow after experimental basal ganglia hemorrhage." Annals of Neurology **11**(3): 266-271.

Rosenberg, G. A., S. Mun-Bryce, M. Wesley and M. Kornfeld (2018). "Collagenase-induced intracerebral hemorrhage in rats." Stroke **21**(5): 801-807.

Sharkey, J., I. M. Ritchie and P. A. Kelly (1993). "Perivascular Microapplication of Endothelin-1: A New Model of Focal Cerebral Ischaemia in the Rat." Journal of Cerebral Blood Flow & Metabolism **13**(5): 865-871.

Tasca, C. I., T. Dal-Cim and H. Cimarosti (2015). "In vitro oxygen-glucose deprivation to study ischemic cell death." Methods Mol Biol **1254**: 197-210.

Watson, B. D., W. D. Dietrich, R. Busto, M. S. Wachtel and M. D. Ginsberg (1985). "Induction of reproducible brain infarction by photochemically initiated thrombosis." Annals of Neurology **17**(5): 497-504.

Yamaguchi, M., J. W. Calvert, G. Kusaka and J. H. Zhang (2005). "One-Stage Anterior Approach for Four-Vessel Occlusion in Rat." Stroke **36**(10): 2212-2214.
