# Supplementary material for: Astrocytes in stroke-induced neurodegeneration: a timeline
Source: Front Mol Med. 2023 Sep 7;3:1240862. doi: 10.3389/fmmed.2023.1240862 (PMC11285566; doi:10.3389/fmmed.2023.1240862)
Supplement: Supplementary file 2 [file Table2.DOCX]

**Table 1**. Astrocytic targets identified in the post-ischemic brain in pre-clinical studies and experimental approach to prevent pathophysiological mechanisms.

| **Astrocytic target** | **Pathophysiological mechanism** | **Experimental approach** | **Species and stroke model** | **References** |
| --- | --- | --- | --- | --- |
| Nrf2 | Oxidative stress | Pharmacological upregulation of astrocytic Nrf2. | Mouse, tMCAO | Hong 2023  Takagi 2014 |
| DJ1 | Oxidative stress | Downregulation of DJ1. | Rat, tMCAO  Mouse, endothelin-1 | Aleyasin 2007  Peng 2019 |
| GLT1 | Excitotoxicity | Pharmacological or genetic upregulation of GLT1. | Rat, tMCAO | Harvey 2011  Chu 2007 |
| VRACs | Excitotoxicity | Tamoxifen inhibition of VRACs  Elimination of Swell1 in astrocytes. | Rat, tMCAO  Mouse, tMCAO | Feustel 2004  Yang 2019 |
| KB | Metabolic imbalance | Diet-induced production of KBs. | Rat, tMCAO | Puchowicz 2008 |
| Mitochondria | Metabolic imbalance | Infusion of exogenous mitochondrial particles into the cerebral cortex. | Mouse, tMCAO | Hayakawa 2016 |
| NHE1 | Edema | Elimination of NHE1. | Mouse, tMCAO | Begum 2018 |
| AQP4 | Edema | Elimination of AQP4 (only beneficial during the acute phase). | Mouse, pMCAO | Zeng 2012  Manley 2000 |
| Ephrin A5 | Reactive astrogliosis | Blocking of EphrinA5 by EphA5-Fc delivery via hydrogel. | Mouse, photothrombosis | Overman 2012 |
| CSPGs | Reactive astrogliosis, axonal growth | Perilesional infusion of chondroitinase to digest CSPGs. | Rat, endothelin-1 | Gherardini 2015 |
| MEGF10  MERTK | Neuroinflammation | Upregulation of MEGF10 and MERTK-mediated signaling. | Mouse, tMCAO | Shi 2021  Morizawa 2017 |
| IL15 | Neuroinflammation | Blocking IL15 with neutralizing antibodies or genetic elimination. | Mouse, tMCAO | Li 2017  Lee 2018 |
| Notch1 | Neurogenesis | Activation of Notch1 signaling. | Mouse, pMCAO | Kraft 2017 |
| SDF1 | Neurogenesis | Increase SDF1 to promote neurogenesis. | Mouse, MCAO | Imitola 2004 |
| ASCL1 | Neurogenesis | Intracortical injection of endothelial-derived microvesicles to deliver ASCL1. | Mouse, tMCAO | Li 2022 |
| Chrdl1 | Synaptic plasticity | Elimination of Chrdl1. | Mouse, photothrombosis | Blanco-Suarez 2022 |
| LZK | Axonal growth | Astrocyte-specific overexpression of LZK. | Mouse, photothrombosis | Chen 2022 |
| Shh | BBB disruption | Intraventricular injection of Shh. | Rat, pMCAO | Xia 2013 |
| P2Y1 | PSCI | Elimination of P2Y1. | Mouse, tMCAO | Chin 2013 |
